# Heterogeneity in myocardial iron content in pediatric and young adult patients

**DOI:** 10.1186/1532-429X-15-S1-P287

**Published:** 2013-01-30

**Authors:** Tim Slesnick, Jeanne Boudreaux, Denver Sallee, WJ Parks

**Affiliations:** 1 Pediatrics, Emory University, Atlanta, GA, USA

## Background

Non-invasive quantification of myocardial iron content in patients with chronic anemia by Cardiac Magnetic Resonance (CMR) using T2* imaging sequences has significantly improved their clinical care. Measurement of myocardial iron content is traditionally performed on the interventricular septum of a single, mid-ventricular short axis image. Limited data exists to evaluate the reproducibility of myocardial T2* imaging in pediatric and young adult patients. Furthermore, there is no data in this patient group evaluating the heterogeneity of myocardial iron throughout the ventricular septum (apex versus mid versus basilar levels).

## Methods

All patients underwent standardized T2* imaging with a multiecho, gradient echo sequence obtained during a single breath hold, with 3 copies of the mid-ventricular slice repeated. The protocol was then expanded to include apical, mid, and basilar level slices, with repeated acquisitions at each location. Retrospective review with re-measurement of all T2* values was performed using a local workstation.

## Results

Ten patients had repeated measurements of the their mid-ventricular T2* imaging, and an additional 8 patients had measurements at three slice locations (9/18 males, median age 19.3 years, range 11.2-23.6 years). For the initial 10 patients, the median T2* value was 21.3 ms (range 6.3-36.0), with a median standard deviation of 1.5 ms (range 0.1-4.4) at the mid-ventricular level. Two of ten patients had values that spanned the clinically accepted cut-point of 20 ms, indicating they could have been classified as either normal or mildly abnormal depending on which image was selected for analysis. For the 8 patients with multiple slice locations analyzed, the median T2* values were 29.5 ms (9.5-47.2), 25.9 (12.5-37.3), and 22.7 (9.9-33.7) for the apex, mid, and basilar levels respectively. While the median standard deviations for each slice location were low (0.6, 0.9, and 0.5 respectively), the differences between the slice locations resulted in 1 patient having values both above and below 20 ms, and 2 additional patients with values above and below the "severely abnormal" cut-point of 10 ms.

## Conclusions

Variability exists in T2* values for both same slice position and different slice positions from apex to basilar interventricular septal myocardial segments in pediatric and young adult patients. Five of 18 patients had values both above and below clinically accepted cut-points for normal, mildly abnormal, and severely abnormal iron content on separate images. This heterogeneity in myocardial iron content implies that a more comprehensive routine myocardial evaluation may be indicated for these patients.

## Funding

None.

